# Geometric Calibration and Validation of Kompsat-3A AEISS-A Camera

**DOI:** 10.3390/s16101776

**Published:** 2016-10-24

**Authors:** Doocheon Seo, Jaehong Oh, Changno Lee, Donghan Lee, Haejin Choi

**Affiliations:** 1 Korea Aerospace Research Institute, Daejeon 34133, Korea; dcivil@kari.re.kr (D.S.); dhlee@kari.re.kr (D.L.); hjchoi@kari.re.kr (H.C.); 2 Department of Civil Engineering, Chonnam National University, Gwangju 61186, Korea; 3 Department of Civil Engineering, Seoul National University of Science and Technology, Seoul 01811, Korea; changno@seoultech.ac.kr

**Keywords:** Kompsat-3A, AEISS-A, calibration, validation

## Abstract

Kompsat-3A, which was launched on 25 March 2015, is a sister spacecraft of the Kompsat-3 developed by the Korea Aerospace Research Institute (KARI). Kompsat-3A’s AEISS-A (Advanced Electronic Image Scanning System-A) camera is similar to Kompsat-3’s AEISS but it was designed to provide PAN (Panchromatic) resolution of 0.55 m, MS (multispectral) resolution of 2.20 m, and TIR (thermal infrared) at 5.5 m resolution. In this paper we present the geometric calibration and validation work of Kompsat-3A that was completed last year. A set of images over the test sites was taken for two months and was utilized for the work. The workflow includes the boresight calibration, CCDs (charge-coupled devices) alignment and focal length determination, the merge of two CCD lines, and the band-to-band registration. Then, the positional accuracies without any GCPs (ground control points) were validated for hundreds of test sites across the world using various image acquisition modes. In addition, we checked the planimetric accuracy by bundle adjustments with GCPs.

## 1. Introduction

Kompsat-3A, which was launched on 25 March 2015, is a sister spacecraft of the Kompsat-3 developed by the Korea Aerospace Research Institute (KARI). Kompsat-3A’s AEISS-A (Advanced Electronic Image Scanning System-A) camera is similar to Kompsat-3’s AEISS but it was designed to provide PAN (Panchromatic) resolution of 0.55 m, MS (multispectral) resolution of 2.20 m, and TIR (thermal infrared) at 5.5 m resolution as presented in Table 1. The altitude of Kompsat-3A is 528 km—which is lower than that of Kompsat-3 (685 km)—for better spatial resolution, sacrificing the swath width. 

In-orbit geometric calibration and validation of high-resolution Earth-observation satellites is important because various impulses and vibrations during the launch process may have affected the satellite’s payload [1,2,3,4,5,6,7,8]. Therefore, the in-orbit geometric calibration process determines the focal length, the distortions of lenses, CCD (charge-coupled device) alignments, and other geometric distortions. For this purpose, bundle adjustments are carried out utilizing GCPs (ground control points) at the reference sites that give accurate location information. This leads to the elimination of a series of systematic errors and reduction of correlation between the interior and exterior orientation parameters to improve the geometric accuracy. Then the validation process is conducted to check and ensure the mapping accuracy.

The geometric calibration and validation of the Kompsat-3A AEISS-A camera have been completed [9]. The geometric calibration consisted of two phases. At phase I, AOCS (attitude and orbit control subsystem) in-orbit calibration was performed with the satellite’s position and attitude data, which are estimated through time synchronization of GPS, AOCS, and payloads. Phase II included the calibration of CCD alignments and the focal length, the CCD overlap area correction, and band-to-band alignments. This was followed by the validation of positional accuracy.

In this paper, we introduce the Kompsat-3A AEISS-A camera and the physical sensor model that incorporates the interior and exterior orientation parameters. Based on the rigorous sensor model, the geometric calibration method of Kompsat-3A will be explained. This includes the boresight calibration, and the calibration of the CCD alignments and the focal length. Then we present the results of the geometric calibration and validation including not only the aforementioned sensor calibrations but the merge of sub-images and the band-to-band registrations. Finally, the positional accuracy after the calibration is presented.

## 2. Kompsat-3A AEISS-A Camera 

### 2.1. AEISS-A Sensor

**Figure 1:**
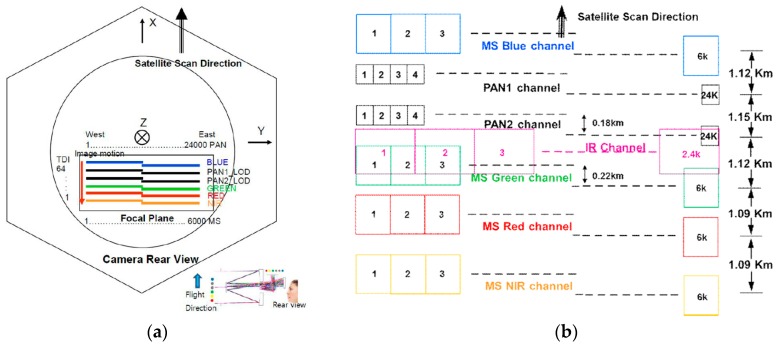
Kompsat-3A AEISS-A (Advanced Electronic Image Scanning System-A) sensor configuration. (**a**) Camera rear view; (**b**) CCD array configurations.

Figure 1a shows the configuration of Kompsat-3A AEISS-A camera. Blue, PAN1, PAN2, TIR, green, red, and near-infrared (NIR) channels are aligned in a unifocal camera. Figure 1b depicts the design of PAN, MS, and TIR (written IR in the figure). The gaps between the sensors in the focal plane correspond to the differences of the projection centers. The telescope uses a Korsch combination with three aspheric mirrors and two folding mirrors, using an aperture diameter of 80 cm. This design was chosen because of its simplicity and compact size, allowing it to fit within the small spacecraft platform. Also, the camera was designed to minimize the aberration.

**Figure 2:**
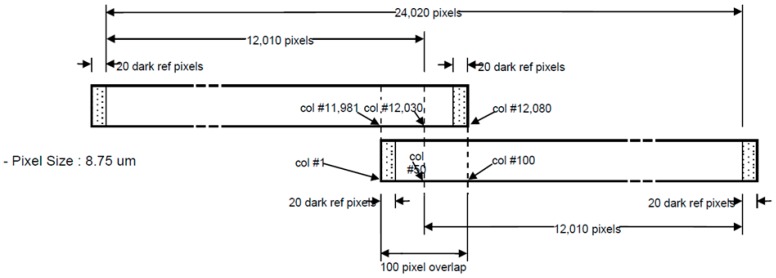
Kompsat-3A AEISS-A panchromatic CCD-lines configuration with an overlapping zone (the scan direction is upward).

Figure 2 presents the detailed configuration of the panchromatic CCD-lines [10]. A single CCD-line consists of 12,080 pixels with 20 dark pixels on each side and the overlapping area is 100 pixels in the center. The pixel size is 8.75 micron. Each CCD-line produces a single subimage with overlapping areas, and the two produced subimages must be merged together into a single image that is 24,020 pixels of image width.

### 2.2. Physical Sensor Modeling

The physical sensor model of Kompsat-3A is in a nonlinear form of projection from a given ground point in an Earth-centered Earth-fixed (ECEF) coordinate frame to a point in the body coordinate frame as shown in Equations (1) and (2). We call this the forward model. The exterior orientation parameters (EOPs) can be interpolated from the ephemeris data given an instant time.

(1) $$\left[\begin{array}{c} U\\ V\\ W \end{array}\right]=M_{Bore}^{T}M_{Orbit}^{Body}M_{ECEF}^{Orbit}\left[\begin{array}{c} X-X_{S}\\ Y-Y_{S}\\ Z-Z_{S} \end{array}\right]$$

(2) $$\left[\begin{array}{c} x_{b}\\ y_{b}\\ z_{b} \end{array}\right]=\lambda \left[\begin{array}{c} U\\ V\\ W \end{array}\right]$$

where ${\left[\begin{array}{ccc} X & Y & Z \end{array}\right]}^{T}$ is the ground point in the ECEF coordinate frame, ${\left[\begin{array}{ccc} X_{S} & Y_{S} & Z_{S} \end{array}\right]}^{T}$ is the satellite position in the ECEF coordinate frame, $M_{ECEF}^{ECI}$ is the time-dependent rotation matrix from the ECEF coordinate frame to the inertial coordinate frame, $M_{ECI}^{Body}$ is the time-dependent rotation matrix from the inertial coordinate frame to the body coordinates frame, $M_{Bore}$ is the boresight rotation matrix, $x_{b},y_{b},z_{b}$ are the coordinates in the body coordinate frame ($x_{b}$ is the flight direction, $z_{b}$ is the direction to the Earth, and $y_{b}$ completes the right-handed coordinate system), and λ is the scale factor.

The position and the rotation of the satellite at time t can be computed using Equation (3). A scan time t corresponding to an image line is used for the computation of the position and the rotation using the Lagrange interpolation of 8 neighboring ephemeris data.

(3) $$\vec{P}(t)=\sum_{j=1}^{8}\vec{P}(t_{j})\times \prod_{\begin{array}{l} i=1\\ i\neq j \end{array}}^{8}\left(t-t_{i}\right)/\prod_{\begin{array}{l} i=1\\ i\neq j \end{array}}^{8}\left(t_{j}-t_{i}\right)$$

where $\vec{P}(t)$ is the position and the rotation of the satellite at time t.

**Figure 3:**
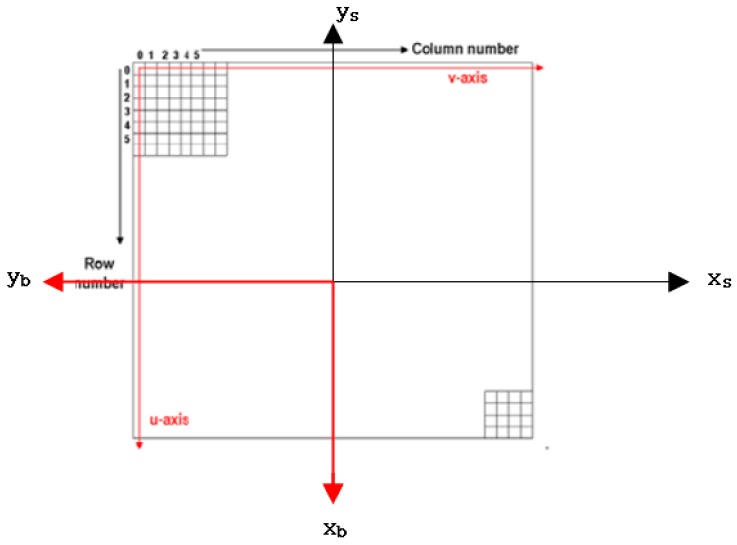
The relationship between the sensor coordinate frame and the body coordinate frame.

The relationship between the sensor coordinate frame and the body coordinates frame is presented in Figure 3 and Equation (4). In, $x_{s},y_{s}$ show the sensor coordinate frame and $z_{s}$ completes the right-handed coordinate system.

(4) $$\left[\begin{array}{c} -y_{s}\\ -x_{s}\\ f \end{array}\right]=M_{b}^{s}\left[\begin{array}{c} x_{b}\\ y_{b}\\ z_{b} \end{array}\right],\;M_{b}^{s}=\left[\begin{array}{ccc} 0 & -1 & 0\\ -1 & 0 & 0\\ 0 & 0 & -1 \end{array}\right]$$

where $M_{b}^{s}$ is the transformation matrix from the body coordinate frame to the sensor coordinate frame, and f is the focal length.

The sensor coordinates can be converted from the image coordinates using CCD-line alignment information parameters as shown in Equation (5). An individual CCD-line requires unique alignment parameters. The CCD alignment equation was determined based on the precise calibration performed before the launch. The second-order equation showed 0.01% difference compared to the reference, satisfying the requirement of distortion 0.2%.

(5) $$\begin{array}{l} x_{s}=a_{0i}+a_{1i}\times \left(c-c_{0i}\right)+a_{2i}\times {\left(c-c_{0i}\right)}^{2}\\ y_{s}=b_{0i}+b_{1i}\times \left(c-c_{0i}\right)+b_{2i}\times {\left(c-c_{0i}\right)}^{2} \end{array}$$

where i is the CCD chip index, $a_{0},b_{0}$ are the x,y coordinates of the first pixel in the CCD chip, $a_{1},a_{2}$ are related to the pixel size ($a_{2}$ is the nonlinearity part), $b_{1},b_{2}$ are the alignment parameters of the non-straight line CCD chip, and c is the column (sample) coordinate in pixels ($c_{0}$ is for the first column of the CCD chip).

### 2.3. Sensor Geometric Calibrations

During the calibration process, the focal length, the boresight angles, and CCD alignment parameters are estimated. Removing the scale factor in Equation (2), observation equations can be established as Equation (6).

(6) $$\begin{array}{l} F_{x}=x_{b}-f\frac{U}{W}=0\\ F_{y}=y_{b}-f\frac{V}{W}=0 \end{array}$$

The first step of the AOCS absolute calibration is to perform the boresight calibration between the star tracker and the other payloads using GCPs (ground control points) located at calibration sites. To this end, the boresight rotation matrix $M_{Bore}$ must be estimated.

(7) $$M_{Bore}=M_{Y_{b}}M_{P_{b}}M_{R_{b}}$$

(8) $$M_{Y_{b}}=\;\left[\begin{array}{ccc} cY_{b} & sY_{b} & 0\\ -sY_{b} & cY_{b} & 0\\ 0 & 0 & 1 \end{array}\right],\;M_{P_{b}}=\;\left[\begin{array}{ccc} cP_{b} & 0 & -sP_{b}\\ 0 & 1 & 0\\ sP_{b} & 0 & cP_{b} \end{array}\right],\;M_{R_{b}}=\left[\begin{array}{ccc} 1 & 0 & 0\\ 0 & cR_{b} & sR_{b}\\ 0 & -sR_{b} & cR_{b} \end{array}\right]$$

(9) $$\begin{array}{l} cY_{b}=\cos(yaw_{B}),\;sY_{b}=\sin(yaw_{B})\\ cP_{b}=\cos(pitch_{B}),\;sP_{b}=\sin(pitch_{B})\\ cR_{b}=\cos(roll_{B}),\;sR_{b}=\sin(roll_{B}) \end{array}$$

The partial derivatives with respect to the boresight angles can be expressed as Equations (10) and (11), which show the case of the boresight roll angle. The same analogy is applied to the other angle cases, such as the pitch and yaw.

(10) $$\begin{array}{l} \frac{\partial F_{x}}{\partial R_{b}}=-\frac{f}{W}\left(\frac{\partial U}{\partial R_{b}}-\frac{U}{W}\frac{\partial W}{\partial R_{b}}\right)\\ \frac{\partial F_{y}}{\partial R_{b}}=-\frac{f}{W}\left(\frac{\partial V}{\partial R_{b}}-\frac{V}{W}\frac{\partial W}{\partial R_{b}}\right) \end{array}$$

(11) $$\frac{\partial }{\partial R_{b}}\left[\begin{array}{c} U\\ V\\ W \end{array}\right]=\left[\begin{array}{ccc} 0 & 0 & 0\\ 0 & -sR_{b} & -cR_{b}\\ 0 & cR_{b} & -sR_{b} \end{array}\right]M_{P_{b}}^{T}M_{Y_{b}}^{T}M_{Orbit}^{Body}M_{ECEF}^{Orbit}\left[\begin{array}{c} X-X_{S}\\ Y-Y_{S}\\ Z-Z_{S} \end{array}\right]$$

Secondly, the calibration of the focal length is simply carried out by deriving the partial derivatives with respect to the focal length as Equation (12).

(12) $$\frac{\partial F_{x}}{\partial f}=-\frac{U}{W},\;\frac{\partial F_{y}}{\partial f}=-\frac{V}{W}$$

Finally, the calibration for the CCD alignment parameters can also be carried out by computing partial derivatives as shown in Equation (13). Note that Kompsat-3A AEISS-A sensor consists of several CCD lines and they are calibrated all together. Note that i is the CCD chip index.

(13) $$\left[\begin{array}{cccccc} \frac{\partial F_{x_{i}}}{\partial a_{0i}} & \frac{\partial F_{x_{i}}}{\partial a_{1i}} & \frac{\partial F_{x_{i}}}{\partial a_{2i}} & \frac{\partial F_{x_{i}}}{\partial b_{0i}} & \frac{\partial F_{x_{i}}}{\partial b_{1i}} & \frac{\partial F_{x_{i}}}{\partial b_{2i}}\\ \frac{\partial F_{y_{i}}}{\partial a_{0i}} & \frac{\partial F_{y_{i}}}{\partial a_{1i}} & \frac{\partial F_{y_{i}}}{\partial a_{2i}} & \frac{\partial F_{y_{i}}}{\partial b_{0i}} & \frac{\partial F_{y_{i}}}{\partial b_{1i}} & \frac{\partial F_{y_{i}}}{\partial b_{2i}} \end{array}\right]=\left[\begin{array}{cccccc} 1 & \left(c-c_{0i}\right) & {\left(c-c_{0i}\right)}^{2} & 0 & 0 & 0\\ 0 & 0 & 0 & 1 & \left(c-c_{0i}\right) & {\left(c-c_{0i}\right)}^{2} \end{array}\right]$$

The partial derivatives with respect to the focal length, the boresight angles, and CCD alignment parameters, as well as EOPs (exterior orientation parameters) are used to form a design matrix A for the linearized observation equation in Equation (14) and iteratively solved using the least square adjustment as Equation (15). Note that the calibrations of boresight angles, focal length, and CCD alignment parameters are carried out sequentially to avoid large correlations among the parameters. Note that single iterative least squares can make the normal matrix not invertible. Therefore, other systems utilized a step-by-step approach for the geometric calibration [11].

(14) Aξ=y

(15) $$\hat{\xi }={\left(A^{T}PA\right)}^{-1}A^{T}Py$$

## 3. Geometric Calibration and Validation

### 3.1. Workflow

**Figure 4:**
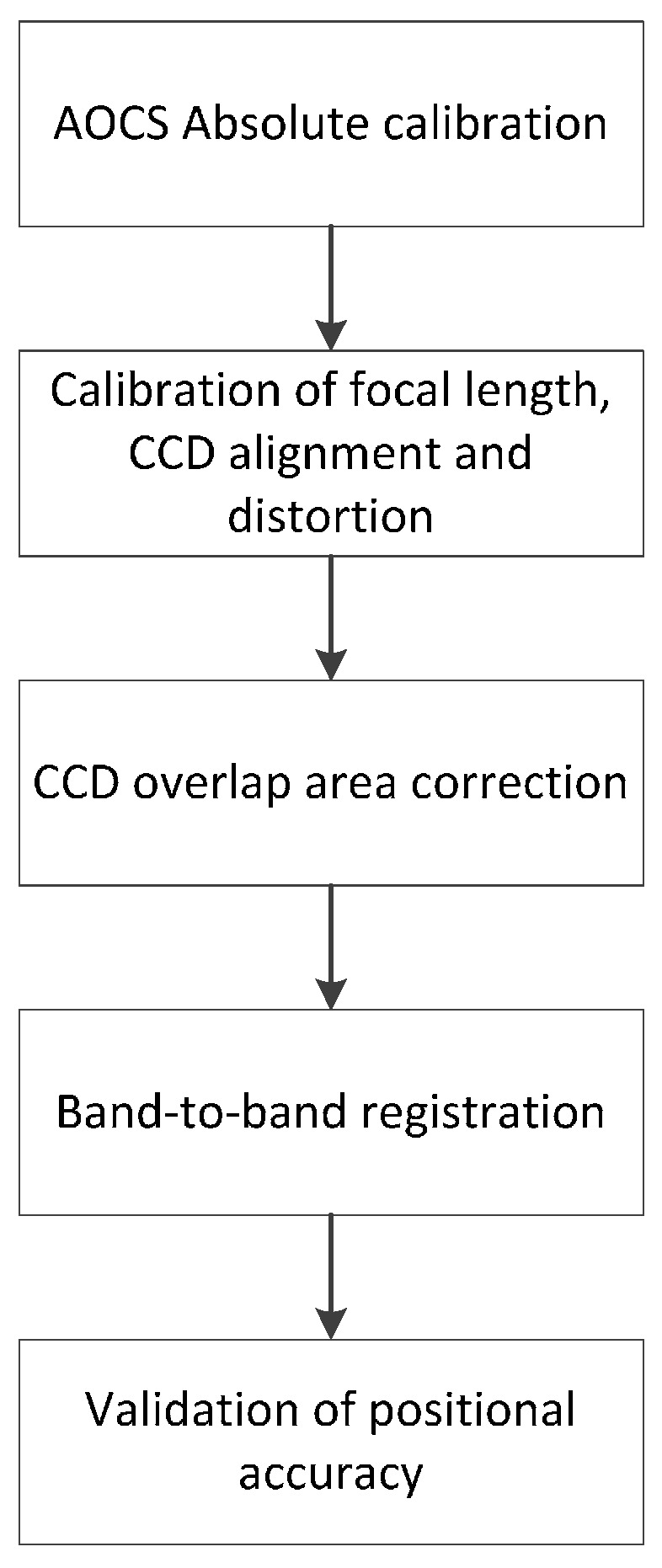
Summarized geometric calibration procedure of Kompsat-3A.

The geometric calibration consists of AOCS in-orbit calibration (boresight calibration), the focal length calibration, the calibration of CCD alignments, the CCD overlap area correction, and band-to-band registration, as shown in Figure 4. The details on the AOCS absolute calibration, focal length, and the CCD alignments were presented in the previous section. For the CCD overlap area correction and band-to-band registration we applied the same merging process used for Kompsat-3 data [10]. This method generates a grid of tie points over the subimages based on the physical sensor model and uses them for similarity transformation with the compensation of ephemeris and terrain variation.

### 3.2. Calibration Sites

We classified test sites into two categories according to their positional accuracies. Level 0 sites are located in several sites over Mongolia and Korea where about 150~180 circle targets of 3 m diameter were established. The coordinates of the targets were acquired by GNSS surveys and they showed the positional accuracy of 5 cm in RMSE (root mean square error). They were used for the CCD alignment, the focal length calibration, AOCS absolute calibration, and validation of mapping accuracy. Average 9~20 points were used for each scene.

**Figure 5:**
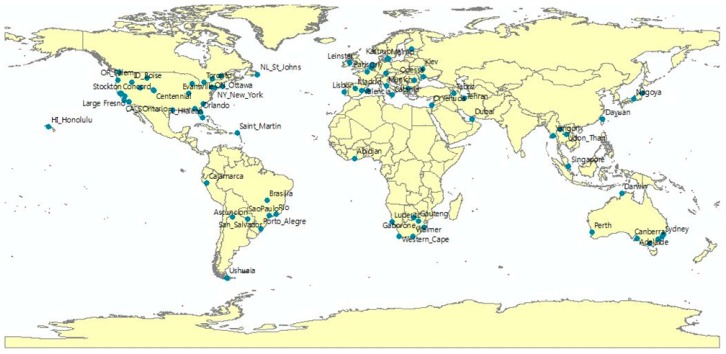
Level 1 site locations.

Level 1 sites are distributed at 82 locations across the world as shown in Figure 5. Locations such as road intersection were global navigation satellite system (GNSS)-surveyed with about 70 cm accuracy in RMSE. Level 1 reference data were used for AOCS absolute calibration and validation of positional accuracy.

### 3.3. AOCS Absolute Calibration 

First we conducted the AOCS absolute calibration. The accuracy of the AOCS absolute calibration highly depends on the accuracy of GCPs, and its reliability can also be affected by the temperature characteristics of the star tracker. Note that the accuracy of the star tracker used (Sodern SED36) is 1 arcsec for the cross-boresight and 6 arcsec for the boresight axis. This necessitated using the GCPs from different calibration sites of the southern and northern hemispheres to carry out the boresight calibration. We used five image strips over level 0 sites and six strips over the level 1 sites with difference roll angles ranging −25.5°~27.9° for the calibration as shown in Table 2.

**Figure 6:**
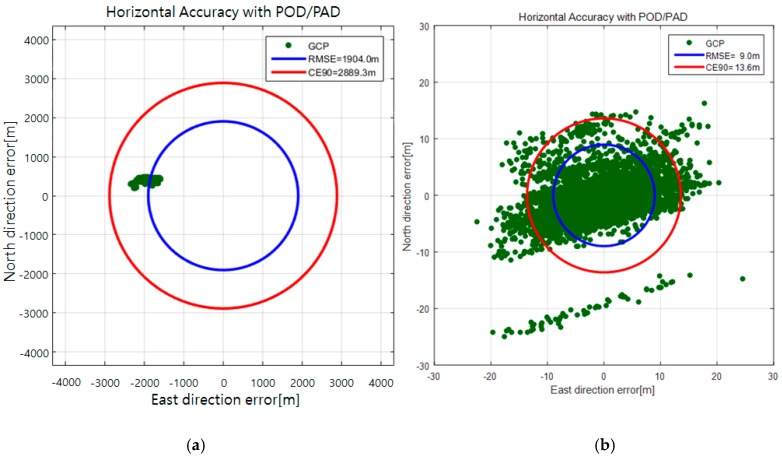
Horizontal accuracy in the ground before (**a**) and after the AOCS calibration (**b**).

The result of the calibration, which is the rotation matrix $M_{Bore}$ in Equation (1), was used to update the system. The horizontal accuracy of the check points was estimated to 2.9 km (CE90) before the calibration, but the error was reduced to 13.6 m (CE90) after the system update, as shown in Figure 6.

### 3.4. Calibration of Focal Length and CCD Alignments 

Focal length and CCD alignments are determined before the satellite launch, but the information may change due to the large acceleration during launch. Therefore, in-orbit calibrations should be carried out for better geometric accuracy.

**Figure 7:**
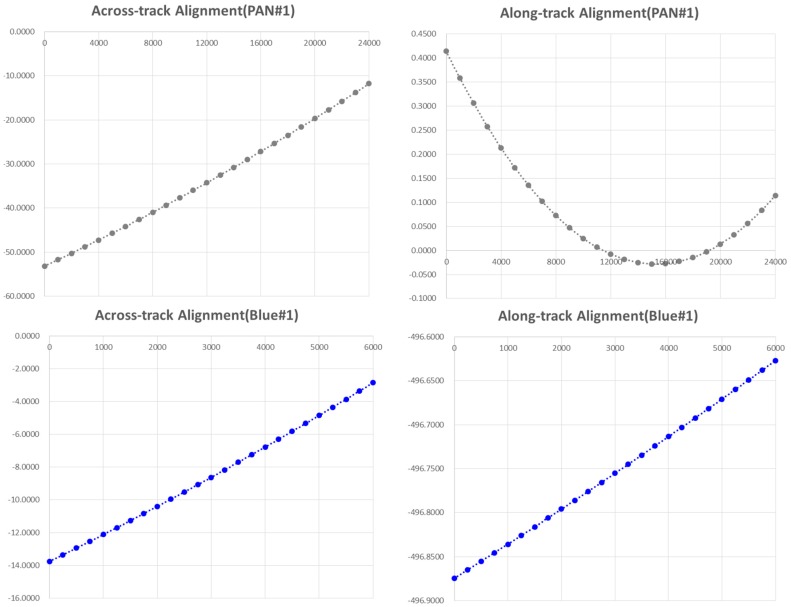
CCD alignment plots for PAN#1 and BLUE#1.

For the in-orbit calibration of focal length and CCD alignment, we utilized 29 images acquired over level 0 calibration sites. The roll and pitch angle ranges are −29.1°~+30.3° and −1.1°~+1.2°, respectively. The focal length of the camera was determined to 8.56181 m and the determined alignment parameters for each CCD sensor are presented in Table 3. Precisions of the calibration for each CCD line are less than one pixel and less than half-pixels for PAN and the others, respectively. Note that the across-track is the direction along the CCD lines and the along-track is opposite to the flight direction as shown in Equation (5). $a_{1}$ and $a_{2}$, linearity, and nonlinearity parameters of the pixel size are determined to be slightly larger than 1.0 and 0.0, respectively, meaning the pixel size is not exactly regular. Also, small but non-zero $b_{1},b_{2}$ values indicate non-straightness of the CCD lines. Thus we plotted the PAN#1 and BLUE#1 CCD lines to see the patterns (Figure 7). PAN #1 shows that the non-regularity of the pixel size accumulates up to about 40 pixels in the across-track alignment. The along-track direction plot shows that the CCD is almost straight with less than half pixels of discrepancy. In case of BLUE#1, the non-regularity of the pixel is accumulated up to about 10 pixels and the non-straightness is about a quarter pixels. 

**Figure 8:**
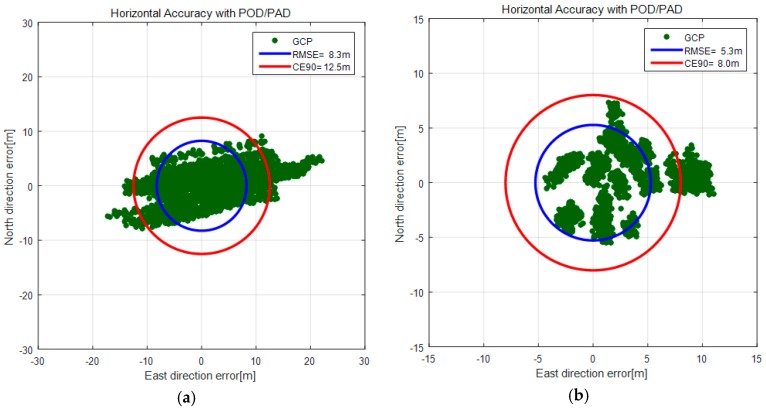
Horizontal accuracy before (**a**) and after the CCD alignment calibration (**b**).

Figure 8 is the plotted horizontal accuracy of GCPs before and after the in-orbit CCD alignment calibration. We can clearly observe the accuracy improvement from 12.5 m (CE90) in Figure 8a to 8.0 m (CE90) in Figure 8b.

### 3.5. Merge of Subimages and Band-to-Band Registration 

**Figure 9:**
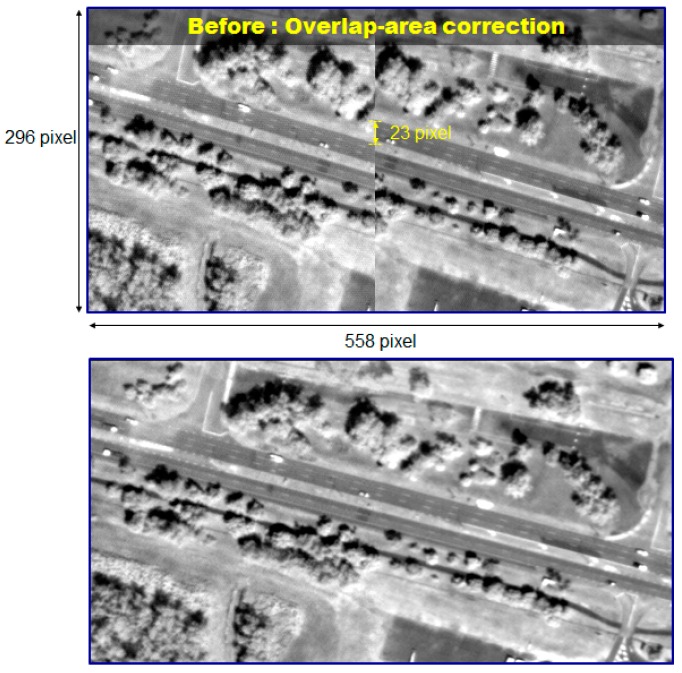
Comparison between before and after the merge of subimages.

As described in Figure 2, individual CCD lines of Kompsat-3A produce overlapping subimages. These subimages should be merged side-by-side for a larger swath width, but the process is not simple because the sensor alignment, ephemeris effects, and terrain elevations should be considered each time. We applied an automated approach using virtual tie points to estimate the shift and similarity transformation, as well as to compute compensations according to the satellite’s attitude differences and terrain elevations due to the gap between the CCD lines [10]. Figure 9 presents an example image before and after the merging. We can hardly identify the discrepancy by applying the process. 

**Figure 10:**
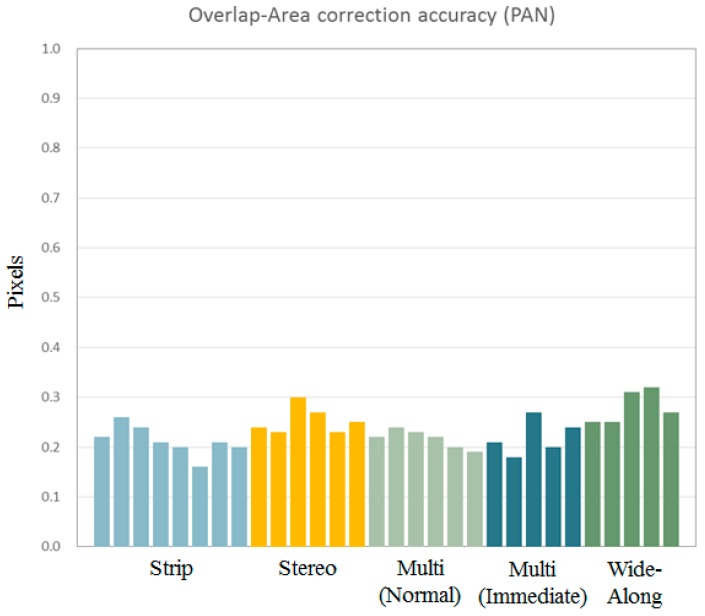
Discrepancy between subimages after the merge for various image acquisition modes.

We tested the quality of the merging results to plot the estimated discrepancies for various acquisition modes of Kompsat-3, as presented in Figure 10. The results showed that the discrepancy between the sub-images from the PAN sensors was estimated to 0.25 pixels (CE90). In addition, the merge quality is not affected by the acquisition modes.

**Figure 11:**
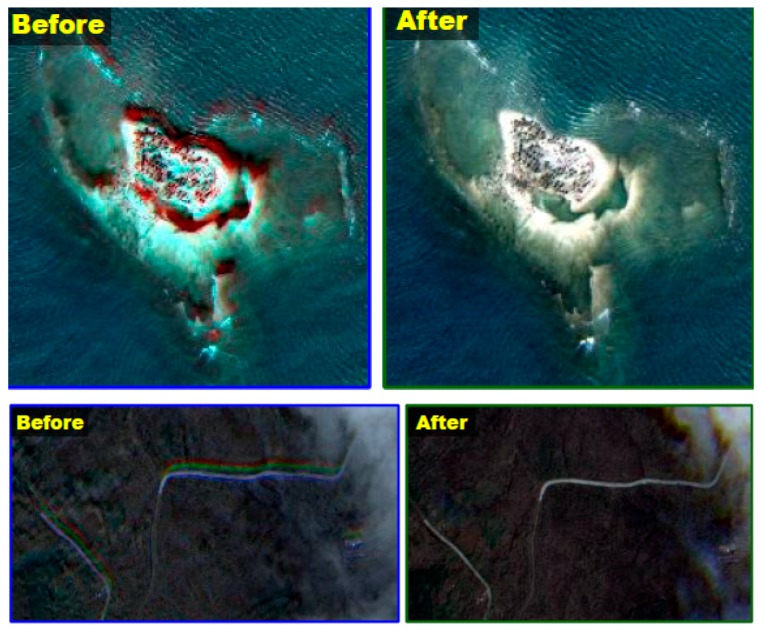
Examples of the band-to-band registration results.

Following the subimage merge, we continue the band-to-band registration process. We utilized a total 30 image strips of various acquisition modes, such as 9 single strips, 6 stereos, 6 standard multi, 5 immediate multi, and 5 wide-along modes. In addition, we used SRTM (shuttle radar topography mission) V2 for the elevation information. Table 4 shows that the accuracy of the registration between PAN and MS sensor is lower than a half pixel in RMSE. Figure 11 shows examples of the band-to-band registration showing the negligible saturation of multispectral colors after the process. 

### 3.6. Validation of Positional Accuracy

Completing the geometric calibration, we validated the positional accuracy. To this end we used 325 sets of test images across the world. The validation data were categorized for several acquisition modes which Kompsat-3A is capable of, as shown in Table 5.

**Figure 12:**
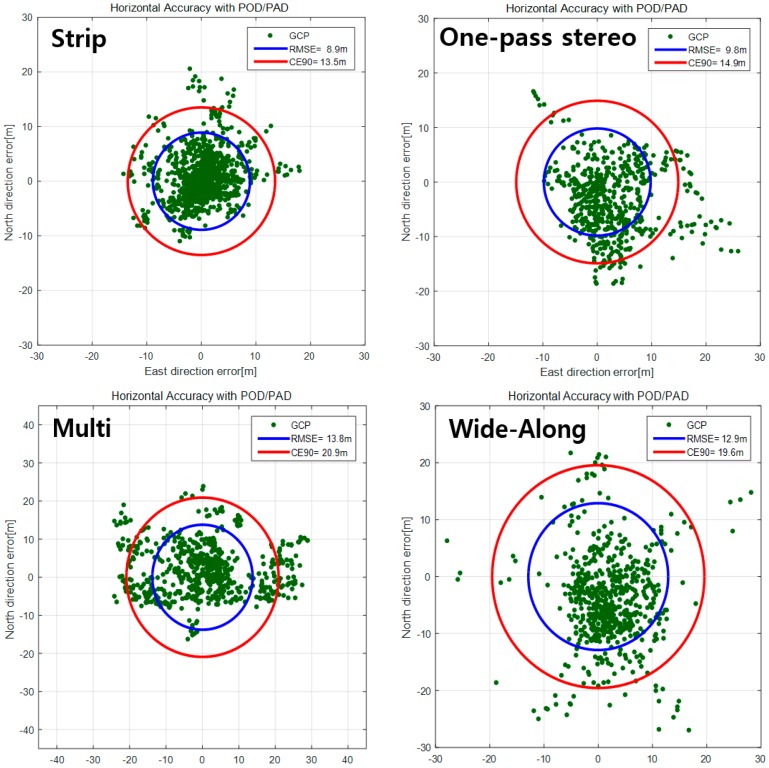
Horizontal accuracy for various acquisition modes without any GCPs.

Figure 12 presents the horizontal accuracy for various image acquisition modes in RMSE and CE90. The strip mode showed the best accuracy among the acquisition modes with 8.9 m (RMSE) and 13.5 m (CE90). The one-pass stereo mode showed about 1 m larger error range than the strip mode. In the cases of multi (normal) and wide-along modes, the accuracy decreased to 13~14 m (RMSE) and 20~21 m (CE90).

**Figure 13:**
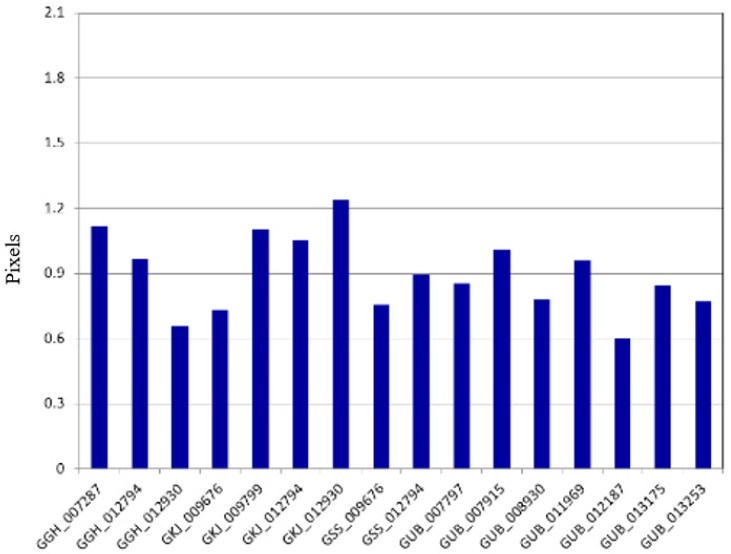
Mapping accuracy for 16 independent data.

Next, we validated the potential mapping accuracy using GCPs. A total of 16 image strips with −21.5°~+29.8° of roll angle range were used for the bundle adjustment. Note that these data were not used for the calibration. Each image was adjusted with 8~9 GCPs and 32~148 check points were used to validate the accuracy. The resultant errors were 0.91 (0.5 m) and 1.39 pixels (0.8 m) in RSME and CE90, respectively, as shown in Figure 13.

## 4. Conclusions

We presented the geometric calibration and validation work of Kompsat-3A that was completed last year. A set of images over the test sites was taken for two months and was utilized for the work. The works include the AOCS absolute calibration, the calibration of the focal length and CCD alignments, the merge of CCD lines, the band-to-band registration, and, finally, by the validation of the positional accuracy.

The successful AOCS’ calibration increased the horizontal accuracy from 2.9 km (CE90) to 13.6 m and the CCD alignment calibration determined the non-regular and nonlinear CCD distortions, improving the accuracy from 12.5 m (CE90) to 8.0 m. Based on the calibration results, we could successfully merge the subimages from each CCD line with a negligible discrepancy of 0.25 pixels (CE90). Finally, we validated the positional accuracy with completion of the geometric calibrations. Without any GCP, the popularly used image acquisition mode, the strip mode, showed 13.5 m of horizontal accuracy, though other modes showed slightly lower accuracies. When GCPs were used for the bundle adjustment, we could obtain less than 1 m of horizontal accuracy in CE90.

## Figures and Tables

**Table 1:** Kompsat-3A specifications.

	PAN (Panchromatic)	MS (Multispectral)
**Spectral Bands**	450–900 µm	Blue: 450–520 µm
		Green: 520–600 µm
		Red: 630–690 µm
		NIR (Near infra-red): 760–900 µm
**GSD (Ground Sample Distance)**	0.55 m at nadir	2.2 m at nadir
**Focal Length**	8.6 m	8.6 m
**Swath Width at Nadir**	12 km	12 km
**Data Quantization**	14 bit	14 bit
**CCD (Charge-Coupled Device) Detector**	Array of 24,000 pixels (2 × 12,000)	Arrays of 4 (RGB and IR) × 6,000 pixels (2 × 3,000)
**Pixel Pitch**	8.75 µm	35 µm

**Table 2:** Attitude angles of Kompsat-3 test data used for the AOCS (attitude and orbit control subsystem) absolute calibration.

Strip	Roll (°)	Pitch (°)	Yaw (°)
1	−15.6	−0.96	2.85
2	0.56	0.20	2.78
3	12.52	0.63	2.09
4	27.91	0.71	1.65
5	0.05	0.17	3.04
6	6.41	0.23	3.33
7	−25.48	−0.99	2.42
8	−9.24	−0.30	2.89
9	27.58	1.16	1.90
10	15.09	0.80	2.31
11	11.88	0.67	2.38

**Table 3:** Determined alignment parameters for each CCD.

Detector	Across-Track (LOD)			Along-Track (LOS)			RMSE
	*a*_0_	*a*_1_	*a*_2_	*b*_0_	*b*_1_	*b*_2_	[pixels]
PAN#1	−12053.14	1.00142	1.26346 × 10^−8^	0.41	−0.00006	1.88813 × 10^−9^	1.00
PAN#2	−12053.07	1.00142	1.25988 × 10^−8^	−340.02	−0.00003	1.96001 × 10^−9^	0.86
Red#1	−3013.24	1.00126	3.50715 × 10^−8^	1556.21	0.00023	−1.25847 × 10^−8^	0.33
Red#2	−3013.16	1.00119	4.51743 × 10^−8^	1454.06	0.00026	−1.34342 × 10^−8^	0.33
Green#1	−3014.48	1.00137	3.73793 × 10^−8^	1058.21	0.00024	−2.70587 × 10^−8^	0.32
Green#2	−3014.41	1.00130	4.71767 × 10^−8^	956.04	0.00029	−2.85802 × 10^−8^	0.32
Blue#1	−3013.74	1.00160	3.68874 × 10^−8^	−496.87	0.00004	5.24967 × 10^−10^	0.32
Blue#2	−3013.68	1.00152	4.80332 × 10^−8^	−599.04	0.00006	1.53648 × 10^−9^	0.28
NIR#1	−3014.57	1.00130	2.56360 × 10^−8^	2052.60	0.00022	−1.60160 × 10^−8^	0.35
NIR#2	−3014.50	1.00125	3.30289 × 10^−8^	1950.17	0.00037	−2.97782 × 10^−8^	0.31

**Table 4:** Band-to-band registration accuracy.

Imaging Type	CalVal_ID	Cloud Level	Scene Center					Average Height (m)	Red-to-PAN		Green-to-PAN		Blue-to-PAN		NIR-to-PAN	
			Latitude	Longitude	Roll	Pitch	Yaw		No	RMSE	No	RMSE	No	RMSE	No	RMSE
Strip	Geo_006839	B	037.7359	−097.1630	30.0	01.4	02.1	416	370	0.23	508	0.22	367	0.27	590	0.22
Strip	Geo_007744	A	−033.4062	−070.5701	−29.8	−01.4	02.2	574	1325	0.19	1307	0.24	1133	0.20	1254	0.19
Strip	GKJ_006331	A	035.8358	126.9800	−29.1	−01.1	02.3	30	1145	0.24	1122	0.24	1035	0.26	1192	0.22
Strip	GSS_006331	A	036.8496	126.6604	−29.1	−01.1	02.2	80	1243	0.18	1284	0.19	1034	0.26	1267	0.25
Strip	GUB_006345	B	047.9453	107.0282	29.8	01.2	01.8	1309	796	0.33	855	0.30	782	0.25	780	0.33
Strip	Geo_006892	C	007.4304	125.9018	29.4	01.6	02.8	304	136	0.30	193	1.18	160	0.58	243	0.39
Strip	Geo_008337	C	045.0623	−093.1176	20.8	01.0	02.2	275	1024	2.81	1022	1.89	1047	0.86	813	4.75
Strip	Geo_005772	B	−012.4020	130.9393	06.7	00.3	03.6	25	869	0.34	873	0.30	721	0.24	909	0.35
Stereo	Geo_006477	B	022.5447	088.4104	−00.4	−30.0	03.2	08	1074	0.25	1263	0.26	1246	0.27	1223	0.28
Stereo	Geo_006478	B	022.5011	088.4200	−00.4	30.2	03.7	07	1076	0.21	1290	0.26	1204	0.26	1194	0.21
Stereo	Geo_008764	B	−035.9403	145.7189	−18.6	−29.4	−07.2	98	228	0.30	377	0.31	323	0.34	1014	0.21
Stereo	Geo_008765	B	−035.9067	145.7085	−18.2	27.6	12.5	102	641	0.36	836	0.34	452	0.41	1063	0.36
Stereo	Geo_009983	B	040.9669	−082.7330	17.5	−27.8	12.0	304	129	0.27	450	0.30	223	0.22	1296	0.24
Stereo	Geo_009984	B	040.9260	−082.7204	17.8	29.5	−07.0	308	240	0.26	542	0.25	401	0.28	1250	0.23
Multi(Normal)	Geo_007676	B	−019.2687	146.8494	28.7	01.4	02.7	26	1179	0.24	1220	0.15	1166	0.19	1192	0.37
Multi(Normal)	Geo_006201	B	−043.5198	172.6841	24.4	00.9	02.3	31	790	0.16	845	0.13	749	0.23	856	0.22
Multi(Normal)	Geo_010131	B	−024.9108	152.4551	28.0	01.3	02.6	24	630	0.34	842	0.22	716	0.25	790	0.44
Multi(Normal)	Geo_008877	B	053.6063	−113.4369	−06.5	−00.1	02.2	672	1124	0.19	1139	0.21	1165	0.25	1313	0.18
Multi(Normal)	Geo_009344	B	047.4359	019.3405	−01.1	00.1	02.5	131	650	0.32	704	0.31	577	0.36	878	0.35
Multi(Normal)	Geo_009502	B	−015.5594	−056.0030	28.1	01.4	02.8	190	1085	0.19	1117	0.09	1108	0.14	1062	0.27
Multi(Immediate)	Geo_002483	B	014.4249	033.6011	−27.5	−01.4	02.8	407	1167	0.20	1150	0.21	1154	0.17	1175	0.28
Multi(Immediate)	Geo_006662	B	−016.7051	−043.7942	02.3	00.0	03.6	672	1033	0.27	1064	0.27	988	0.26	996	0.18
Multi(Immediate)	Geo_007785	B	−027.1914	151.3482	28.3	01.3	02.6	341	767	1.01	822	0.72	866	0.55	848	1.05
Multi(Immediate)	Geo_008606	B	053.5697	−113.3945	26.7	01.0	01.7	667	1282	0.19	1147	0.16	1211	0.12	1157	0.15
Wide-Along	Geo_006826	B	040.7115	−076.6778	11.2	−28.9	08.8	230	136	0.22	366	0.29	190	0.25	672	0.27
Wide-Along	Geo_006827	B	040.7103	−076.5101	13.0	30.0	−04.3	280	178	0.36	474	0.14	311	0.12	594	0.38
Wide-Along	Geo_010468	B	044.3926	−100.2630	−12.2	−29.7	−04.0	492	369	0.37	628	0.29	528	0.33	1213	0.31
Wide-Along	Geo_010469	B	044.3308	−100.4222	−13.8	28.8	10.0	520	66	1.93	392	0.35	102	0.50	758	0.30
Wide-Along	Geo_009221	B	035.6860	−000.5169	28.1	−25.2	17.2	118	1303	0.24	1288	0.21	1220	0.23	1215	0.27

**Table 5:** Test data used for the positional accuracy validation.

Acquisition Mode	Number of Data Sets
**Strip**	94
**Multi (Immediate/Normal)**	63/63
**Wide-Along**	63
**Along-Track Stereo**	56
